# Multifunctional Meta-Aramid Fabrics Enhanced with Inherent Flame-Retardant Polyurea Coatings: Integration of Mechanical Strength, Puncture Resistance, and Self-Healing Properties

**DOI:** 10.3390/polym17111529

**Published:** 2025-05-30

**Authors:** Kang Yang, Yuncheng Zhao, Ke Shang, Bin Zhao

**Affiliations:** 1 Tianjin Key Laboratory of Fire Safety Technology, Tianjin 300381, China; 2 Qingdao Key Laboratory of Flame-Retardant Textile Materials, National Engineering Research Center for Advanced Fire-Safety Materials D & A (Shandong), Institute of Functional Textiles and Advanced Materials, College of Textiles & Clothing, Qingdao University, 308 Ningxia Road, Qingdao 266071, China; 15969693915@163.com (K.Y.); a15831266186@163.com (Y.Z.); 3 Tianjin Fire Science and Technology Research Institute of MEM, Tianjin 300381, China

**Keywords:** flame retardant, polyurea, aramid fiber, mechanical property

## Abstract

In this study, a solvent-free, slow-curing, inherently flame-retardant polyurea coating was successfully developed through an optimized formulation. The novel polyurea was synthesized using mixed Schiff base latent curing agents derived from terminal polyether amines with different-number average molecular weights (D2000 and D400), methyl isobutyl ketone, and polyethyl phosphate glycol ester (OP550). Subsequently, polyurea/meta-aramid (PUA/AF) composite fabrics were fabricated via a scraping coating technique. Thermogravimetric analysis revealed enhanced char formation and reduced decomposition temperatures due to the incorporation of OP550. Comprehensive flame retardant performance was demonstrated through vertical flame testing, limiting oxygen index, and micro-scale combustion calorimetry, with results showing significantly reduced heat release rates, lower total heat release, and increased residual char. Mechanical evaluations indicated marked improvements in tearing, tensile, single-yarn tensile, and bursting forces, attributed to strong fiber–polyurea interfacial interactions, as confirmed by detailed SEM morphological analyses. Moreover, the PUA/AF composites exhibited excellent static puncture resistance and effective self-healing capability. Collectively, these advancements highlight the potential of PUA/AF composite fabrics as promising candidates for advanced protective textiles, integrating superior flame retardancy, mechanical strength, puncture resistance, and self-repairing functionality.

## 1. Introduction

Aramid fibers (AFs) have become indispensable materials for protective textiles due to their high performance and low density [1]. Aramid fibers are generally categorized into para-aramids (e.g., Kevlar), known for their high tensile strength and modulus, and meta-aramids (e.g., Nomex), which provide excellent thermal stability and inherent flame resistance [2]. However, the low inter-fiber friction and limited bonding between yarns in woven fabrics often lead to delamination under localized stress, resulting in poor puncture resistance [3]. This limitation significantly restricts their utility in demanding environments that require both mechanical robustness and reliable protection against sharp or concentrated impacts.

To address this challenge, various surface modification strategies have been employed to improve inter-yarn bonding in AF. Techniques involving shear-thickening fluids (STFs), polymer resins, hydrogels, and other functional coatings have shown considerable potential in enhancing the mechanical strength and puncture resistance of aramid fabrics. Since 2003, STFs have been widely studied as a promising solution for improving the impact and puncture resistance of AF [4]. Subsequent research has explored diverse processing techniques and compositional optimizations, demonstrating that impregnation methods, nanoparticle size, and concentration-dependent formulations critically influence reinforcement effects. Common nanoparticles used in STF systems include nano-silica (SiO_2_) [5,6], multi-walled carbon nanotubes [7], and halloysite nanotubes [8]. Despite their effectiveness, STF-based treatments often compromise fabric breathability and are prone to humidity-induced rheological degradation, necessitating more stable modification strategies.

More recently, resin-based composite technologies have emerged as a mainstream approach for enhancing the puncture resistance of fabrics. By infiltrating resin matrices into fabric interstices, these methods reduce yarn slippage and enable more efficient load distribution across the fiber network, thereby improving energy absorption during impact events [9]. Resins are typically classified into thermosetting and thermoplastic types. Thermosetting resins—such as epoxy, phenolic, and unsaturated polyester—are widely used in rigid composites for their excellent mechanical and thermal performance [10]. Thermoplastic resins—including polyethylene, polypropylene, polyurethane, and Surlyn—offer superior flexibility, and studies have demonstrated their good anti-puncture performance in aramid fiber systems [11,12]. Nonetheless, achieving a balance between mechanical protection, material flexibility, and processing convenience remains a challenge. High curing temperatures required for thermosetting systems, limited post-impact energy absorption in thermoplastic composites, and the inherent flammability of polymer matrices highlight the urgent need for advanced resin systems. Polyimide (PI) coatings can also impart excellent thermal stability and fire protection to AF. However, their application is limited by the need for high-temperature curing, the use of organic solvents, inherent brittleness, and the lack of self-healing capability [13,14].

In this context, polyurea has attracted attention as a protective coating material due to its outstanding waterproofing, corrosion resistance, and impact toughness, making it suitable for applications such as waterproofing, explosion-proofing, and bulletproofing [15,16]. Recently, several studies have investigated polyurea coatings for aramid-based textiles, demonstrating their effectiveness in improving energy dissipation and structural reinforcement under high-impact conditions. The polyurea-coated AF fabrics have been developed for ballistic and blast-resistant applications [17,18], suggesting the potential of polyurea coatings to enhance the puncture resistance of aramid fibers.

However, the rapid reaction kinetics of conventional aromatic polyurea significantly hinder its processability and broader applicability [19,20]. To address this, Zhang et al. [3] developed a scalable, one-component polyurea coating using aldehyde-mediated amino modification, enabling room-temperature self-healing and improved mechanical and puncture performance for Kevlar fabrics. These findings highlight the versatility of polyurea as a multifunctional coating for technical textiles. Despite these advances, polyurea suffers from inherently poor flame retardancy, limiting its suitability for fire-protective textiles. Although flame retardants can be added to improve fire resistance, they often compromise the mechanical properties of the polymer [21,22,23]. Thus, achieving a synergistic balance of flame retardancy, mechanical strength, and puncture resistance in polyurea-coated AF remains a significant challenge.

In our previous study, we developed a solvent-free, intrinsically flame-retardant, slow-curing, and mechanically robust polyurea using a Schiff base latent curing agent (D2000-MIBK) and a phosphate-containing polyol (OP550) [24]. However, the resulting material lacked segmental mobility and thus exhibited no self-healing capability. To overcome this limitation, we now propose an improved formulation by incorporating mixed Schiff base latent curing agents derived from polyether amines of different molecular weights (D400 and D2000). This design, inspired by recent structural optimization strategies [25], enables the formation of a dual-hydrogen-bonded network, D400 forming rigid, high-density hydrogen bond regions for strength, and D2000 contributing flexible, low-density regions for dynamic chain motion and room-temperature self-healing. Simultaneously, the increased OP550 content was introduced to meet the higher flame-retardancy demands of thin polyurea coatings on meta-aramid fabrics. In this study, we specifically focus on meta-aramid fabric, owing to its widespread use in fire-resistant garments and its ability to withstand high-temperature environments [26]. The resulting polyurea/meta-aramid (PUA/AF) composite fabrics, fabricated via a scraping coating process, exhibit simultaneous improvements in flame retardancy, mechanical properties, puncture resistance, and thermally induced self-healing performance. This work presents a rational formulation strategy and a scalable coating process for developing multifunctional protective fabrics with enhanced durability and safety.

## 2. Experiment

### 2.1. Materials

Diethyltoluenediamine (E100, 98%), dimethylthiotoluenediamine (E300, 99.5%), isophorone diisocyanate (IPDI, 99%), poly(propylene oxide) diamine (polyether amine D400, M_n_ = 400), poly(propylene oxide) diamine (polyether amine D2000, M_n_ = 2000), methyl isobutyl ketone (MIBK, 99%), toluene (99.5%), isopropyl alcohol (99.5%), di-n-butylamine (99%), anhydrous sodium carbonate (99.5%), sodium chloride (99.5%), bromocresol green indicator (pH range: 3.8–5.4), bromocresol green–methyl red mixed indicator, concentrated hydrochloric acid (36%), and concentrated sulfuric acid (38%) were purchased from Shanghai Macklin Biochemical Technology Co., Ltd. (Shanghai, China). Polyethyl phosphate glycol ester (commercial name: Exolit OP550, manufactured by Clariant) was supplied by Shanghai King Chemicals Co., Ltd. (Shanghai, China). Meta-aramid fabric (Nomex^®^ fiber from Dupont, plain weave woven fabric, 210 g/m^2^, 0.38 mm thickness) was commercially obtained from Hubei Jiateng textile Co., Ltd. (Xianning, China).

### 2.2. Preparation of Polyurea

#### 2.2.1. Preparation of Terminated Polyether Amine D400-MIBK and D2000-MIBK

The preparation of the terminally modified polyether amine D2000-MIBK was carried out according to previous study [24]. The synthesis of D400-MIBK was slightly modified based on this method. Specifically, a 250 mL three-necked flask was equipped with a constant-pressure dropping funnel and a thermometer. Methyl isobutyl ketone (MIBK, 75 g) was added to the flask, and the temperature was raised to 100 °C. Then, anhydrous polyether amine D400 (100 g) was added dropwise, maintaining a molar ratio of ketone to amine at 3:1. After the addition, the temperature was increased to 105 °C and held for 1 h to allow the reaction to proceed. Subsequently, a Dean–Stark apparatus replaced the dropping funnel, and the flask was again filled with MIBK. The system was then heated to 200 °C to reach the azeotropic temperature to remove water. Once no further phase separation was observed in the Dean–Stark apparatus, the reaction mixture in the flask was subjected to vacuum distillation. The final product, terminally modified polyether amine D400-MIBK, was thus obtained.

#### 2.2.2. Fabrication of the Polyurea/Aramid Composite Fabric

**Scheme 1:**
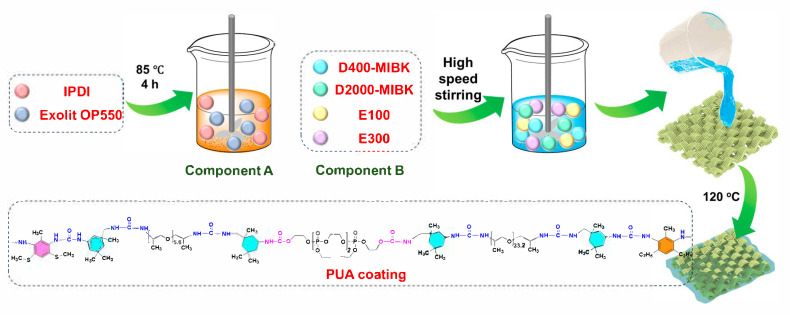
The fabrication process of PUA/AF composite fabric.

The fabrication process comprised three sequential stages as illustrated in Scheme 1:

(1) Synthesis of Polyurea Prepolymer (Component A)

Exolit OP550 was charged into a pre-dried three-necked flask. Controlled dehydration was performed at 120 °C under reduced pressure (−0.095 MPa) for 3 h. After cooling to 85 °C, IPDI was introduced under continuous nitrogen purge to initiate step-growth polymerization. The real-time monitoring of—NCO concentration via dibutylamine titration confirmed reaction progression. Polymerization was terminated when the experimental—NCO content attained 98.0 ± 0.5% of theoretical value. The resultant amber prepolymer was nitrogen-blanketed for storage.

(2) Formulation of Curing System (Component B)

Predetermined quantities of E100, E300, and terminated polyether amines (D2000-MIBK/D400-MIBK) were metrologically dispensed according to Table 1. Homogenization was achieved through mechanical agitation (500 rpm, 30 min), followed by quiescent degassing to yield a bubble-free curing agent.

(3) Composite Integration

Prior to coating, the meta-aramid fabrics were pre-washed with ethanol and deionized water to remove any residual processing finishes, oils, or contaminants that might affect the fiber–matrix adhesion. After cleaning, the fabrics were dried at 60 °C for 1 h to ensure surface readiness for coating. A pretreated plain-woven aramid textile substrate (30 × 30 cm^2^) was surface treated with freshly prepared polyurea solution. Component A and B were volumetrically combined under ambient conditions using high-shear mixing (2000 rpm, 60 s). The immediate PUA coating deposition on AF was executed via a precision coating bar, followed by thermal curing in a convection oven (120 °C, 6 h). This protocol produced PUA/AF composite fabric samples with controlled coating thickness (0.12 ± 0.03 mm). The PUA system used in this study was formulated with stoichiometrically balanced reactive components and thermally cured for 6 h to promote complete conversion.

### 2.3. Characterization

Thermogravimetric profiles were acquired using a TGA 5500 system (TA Instruments, New Castle, DE, USA) with ~3–5 mg specimens under a nitrogen purge (50 mL·min^−1^). Thermal decomposition was monitored from 40 °C to 800 °C at a heating rate of 10 °C·min^−1^.

The vertical flame testing (VFT) was conducted in accordance with GB/T 5455-2014 [27], using 300 × 89 mm^2^ specimens in a TTech-GBT 2408 vertical combustion chamber (TESTech Instrument Technology Co., Ltd., Suzhou, China).

Limiting oxygen index (LOI) values were measured for 150 × 58 mm^2^ strips using a TTech-GBT 2406-1 analyzer (same manufacturer) according to GB/T 5454-1997 [28].

Microscale combustion calorimetry (MCC) was performed on a FAA Micro Calorimeter (Fire Testing Technology Ltd., East Grinstead, UK) in compliance with ASTM D7309-2007 [29]. Approximately 5 mg of sample was thermally decomposed in a gas mixture containing 20% oxygen and 80% nitrogen by volume. The temperature program was executed under dynamic heating at 1 °C·s^−1^, ranging from 40 °C to 750 °C. All thermal tests were performed in triplicate, and average values were reported to ensure data reproducibility.

Tearing properties were tested using the MESDAN-2516 system (MESDAN S.P.A, Peugnago del (Bs), Italy), referencing BS 3424-1982 [30]. According to the Method 7A, at least five rectangular specimens with rectangular tongues should be cut for testing, the power-operated at a rate of 100 ± 10 mm·min^−1^.

Tensile properties were evaluated using a MESDAN electronic textile tester (MESDAN S.P.A, Peugnago del (Bs), Italy) according to GB/T 3923.1-2013 [31] (equivalent to ISO 13934-1:2013 [32]). Specimens (300 × 50 mm^2^) were clamped with a 200 ± 1 mm gauge length and pulled to failure at 100 mm·min^−1^.

Single-yarn tensile force was measured at 300 mm·min^−1^ using a MESDAN-2516 tensile tester. Yarn segments (250 mm in length) were tested in accordance with ASTM D2256/D2256M-21 [33].

Burst resistance was tested with a MESDAN-2516 electronic textile tester using a 25 mm diameter hemispherical plunger at 300 mm·min^−1^, following GB/T 19976-2005 [34] on 100 × 100 mm^2^ samples.

Static puncture resistance was measured on 50 × 50 mm^2^ square specimens using a Instron-3365 multifunctional textile tester (Instron, Boston, MA, USA) equipped with a 3.0 mm diameter hemispherical plunger, operated at 50 mm·min^−1^ in accordance with GB/T 19976-2005.

Artificial scratches were introduced on specimen surfaces using surgical blades. Scratched regions were thermally treated at 80 °C, and healing effectiveness was monitored via optical inspection.

Fracture surfaces, healing interfaces, yarn morphologies, and char residues were imaged using a JSM-6390LV scanning electron microscope (JEOL, Tokyo, Japan) operated at 15 kV.

## 3. Results and Discussion

### 3.1. Thermal Stability

**Figure 1:**
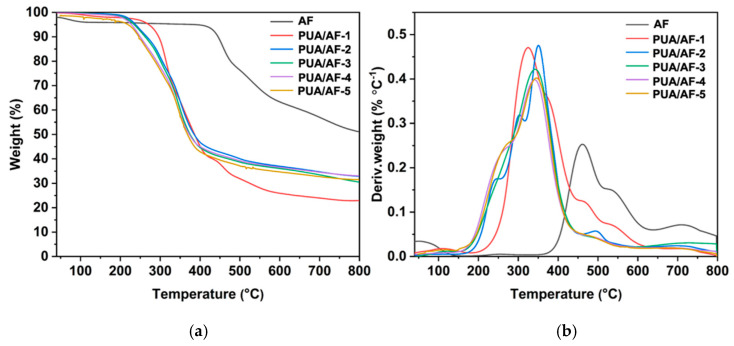
TGA (**a**) and DTG (**b**) curves of the composite fabric samples in a N_2_ atmosphere.

The thermogravimetric analysis (TGA) and derivative thermogravimetry (DTG) curves of the composite fabric samples under a nitrogen atmosphere are presented in Figure 1, with detailed thermal parameters summarized in Table 2. Pure AF exhibited excellent thermal stability, with a 5 wt% weight loss temperature (T_5%_) of 387.3 °C and a peak decomposition temperature (T_max_) of 461.1 °C. This remarkable performance is attributed to the inherent thermal stability of the aromatic polyamide structure, which facilitates robust char formation at 800 °C [35]. The resulting char layer served as an effective thermal barrier, inhibiting further degradation. Upon modification with flame-retardant PUA coatings, the PUA/AF composite fabrics displayed a notable reduction in T_5%_ compared to pure AF, indicating that the PUA coatings begin decomposing at lower temperatures due to the presence of thermally less stable urethane and urea linkages. Specifically, the PUA/AF-1 sample (without OP550) showed a T_5%_ of 271.3 °C, reflecting the intrinsic decomposition behavior of unmodified polyurea. As OP550 was incorporated (PUA/AF-2 to PUA/AF-5), T_5%_ further decreased from 238.1 °C to 218.9 °C, demonstrating that the flame-retardant additive promoted earlier thermal degradation [36]. Interestingly, the T_max_ of the PUA/AF composites initially increased with OP550 addition—from 324.4 °C (PUA/AF-1) to 351.1 °C (PUA/AF-2)—suggesting enhanced dehydration and char formation mechanisms that stabilize intermediate decomposition products. Subsequent formulations (PUA/AF-3 to PUA/AF-5) showed slightly reduced T_max_ values (ranging from 340.2 °C to 345.8 °C), likely indicating a balance between thermal stabilization and the increased presence of thermally labile OP550. Despite the earlier onset of decomposition, the char residue at 800 °C increased significantly with increased OP550 content: from 22.9 wt% in PUA/AF-1 to 32.9 wt% in PUA/AF-5. This substantial rise in residual char underscores the effectiveness of OP550 in promoting char formation at elevated temperatures, thereby enhancing the flame-retardant characteristics of the composite. Overall, the TGA results reveal a clear positive correlation between OP550 content and char forming capability, highlighting its critical role in improving the thermal protection of PUA/AF composite fabric systems.

### 3.2. Flame Retardancy

**Figure 2:**
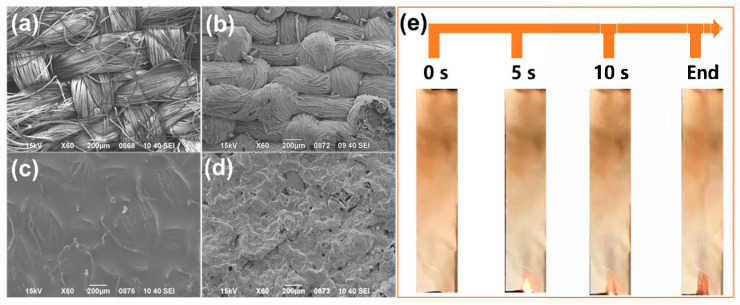
SEM micrographs of the fabrics before vertical flame testing ((**a**) for AF; (**c**) for PUA/AF-5) and its post-test morphologies ((**b**) for AF; (**d**) for PUA/AF-5). Video captures of PUA/AF-5 during vertical flame testing (**e**).

The flame retardancy of the composite fabrics was further assessed using vertical flame testing (VFT) and limiting oxygen index (LOI) measurements, with results summarized in Table 3 and visualized in Figure 2. Pure AF fabric exhibited superior flame resistance, demonstrated by an LOI value of 31.4%, and minimal char length (0.5 cm) with no after-flame and after-glow time. However, PUA/AF-1, being inherently flammable of unmodified PUA, led to rapid flame spread and significant after-flame duration of 16 s. The introduction and progressive increase in OP550 in PUA coatings significantly enhanced the flame retardancy of composite fabrics. As OP550 content rose from PUA/AF-1 to PUA/AF-5, LOI values gradually improved (from 20.9% to 27.8%), and after-flame times reduced, reaching self-extinguishing behavior in PUA/AF-5. The visual captures during the VFT (Figure 2e) clearly depict rapid self-extinguishing properties of PUA/AF-5, with minimal damage progression after initial ignition. The char length was limited to only 3.2 cm, demonstrating effective suppression of flame propagation. Among all formulations, PUA/AF-5 showed the best performance due to its highest OP550 content. The increased phosphorus not only catalyzes early char formation but also facilitates the creation of an intumescent and compact char layer. This barrier improves thermal insulation and oxygen shielding, leading to the observed self-extinguishing behavior and highest LOI. In detail, SEM micrographs in Figure 2 further illustrate the obvious different of AF and AF coated with PUA, demonstrating effects of OP550 on protective char formation, in agreement with TGA results. Before flame exposure, as shown in Figure 2c, PUA coatings uniformly covered the aramid fibers, forming a protective layer. The good adhesion and combination of the coating and fabric can be attributed to both fabric pre-treatment and strong adhesive force of polyurea [37]. After vertical flame testing, PUA/AF-5 exhibited a dense, continuous, and compact char layer, which protected the inner fabrics. This promotes char formation in the polyurea coating, creating a dense and robust barrier that significantly reduces thermal decomposition rates, thereby effectively enhancing the flame retardancy of the composite fabrics.

**Figure 3:**
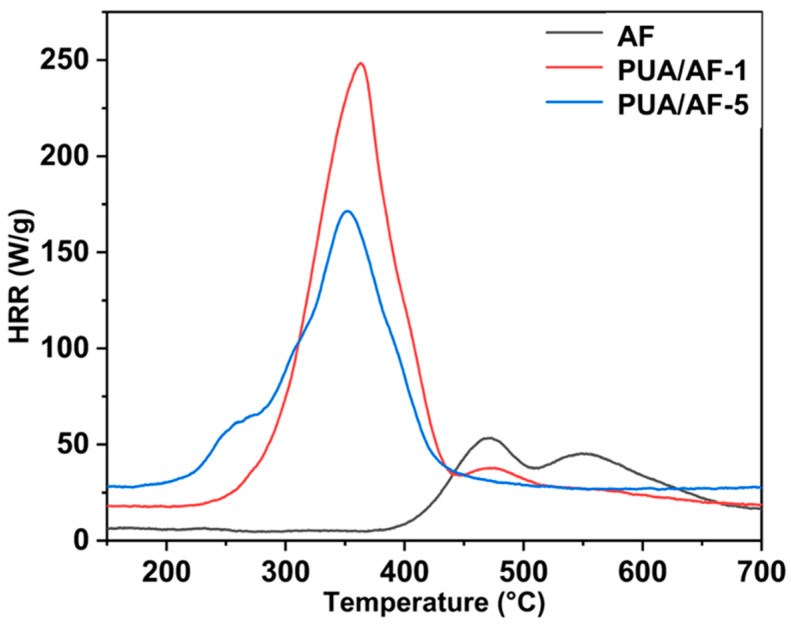
HRR curves of AF, PUA/AF-1, and PUA/AF-5.

Microscale combustion calorimetry (MCC) was performed to further evaluate the combustion behavior of composite fabrics, which provides a reliable quantitative approach to assess the flame-retardant properties of materials with milligram-level sample quantities [38,39]. The resulting heat release rate (HRR) curves and char residue images are presented in Figure 3, with detailed MCC parameters summarized in Table 4. The peak heat release rate (PHRR), which reflects the intensity of heat released during combustion, provides key insight into flammability [40,41]. Pure AF exhibited the lowest PHRR value of 41.1 W·g^−1^, consistent with its inherent flame resistance. In contrast, the PUA-coated fabric without OP550 (PUA/AF-1) showed a markedly higher PHRR of 232.7 W·g^−1^, indicating the high flammability of unmodified polyurea. Notably, the incorporation of OP550 in PUA/AF-5 reduced the PHRR substantially to 144.3 W·g^−1^, demonstrating the flame-retardant effect of the additive. Total heat release (THR) results further confirmed this trend: PUA/AF-5 exhibited a 36% decrease in THR compared to PUA/AF-1 (14.4 vs. 22.4 kJ·g^−1^). These reductions clearly demonstrate the effectiveness of OP550 in suppressing heat release during combustion. The heat release capacity (HRC), which quantifies a material’s tendency to release heat upon decomposition, also showed a significant decrease—from 244 J·g^−1^·K^−1^ in PUA/AF-1 to 145 J·g^−1^·K^−1^ in PUA/AF-5—further confirming the improved fire performance achieved through OP550 incorporation. In addition, the post-combustion char residues supported the MCC findings. PUA/AF-1 yielded a char residue of 21 wt%, whereas PUA/AF-5 achieved a much higher value of 39 wt%. Collectively, these results demonstrate that although the polyurea coating alone increases the heat release potential of the composite fabric, the addition of OP550 effectively mitigates this drawback and significantly improves the overall flame retardancy of the material.

### 3.3. Mechanical Properties

**Figure 4:**
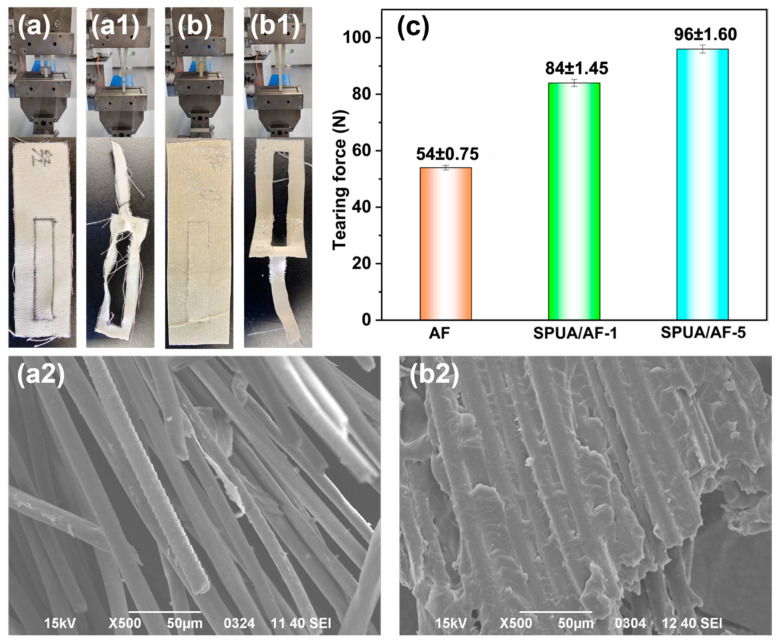
Digital photos of composite fabric before and after tearing tests: AF (**a**,**a1**), PUA/AF-5 (**b**,**b1**); The tearing force of the composite fabric samples (**c**). SEM images of AF (**a2**) and PUA/AF-5 (**b2**) after tearing tests.

The tearing force of the composite fabrics was systematically evaluated, and the corresponding results are presented in Figure 4. During the tearing process, the fabrics underwent pronounced structural deformation characterized by fiber pull-out, interlacing, and extensive yarn extraction, i.e., mechanisms that contribute significantly to energy dissipation. As illustrated in Figure 4c, the PUA-coated fabrics exhibited notable improvements in tearing force compared to the pure AF fabric. The tearing force increased from 54 ± 0.75 N (AF) to 84 ± 1.45 N (PUA/AF-1), and further to 96 ± 1.60 N (PUA/AF-5). A similar enhancement was observed in the weft direction, where the tearing force improved from 58 N (AF) to 65 N (PUA/AF-1), reaching a peak value of 103 N in PUA/AF-5.

Digital photographs and SEM images of the AF fracture surface reveal a loose and disordered fiber structure, as shown in Figure 4(a1,a2), indicative of weak inter-fiber cohesion. In contrast, PUA/AF-5 (Figure 4(b1,b2)) exhibited a more compact fracture morphology, with strong interfacial bonding between fibers and the polyurea matrix. Although some fiber bundles fractured under severe tearing forces, the tight fiber–matrix adhesion enhanced energy dissipation, resulting in significantly improved tearing resistance.

**Figure 5:**
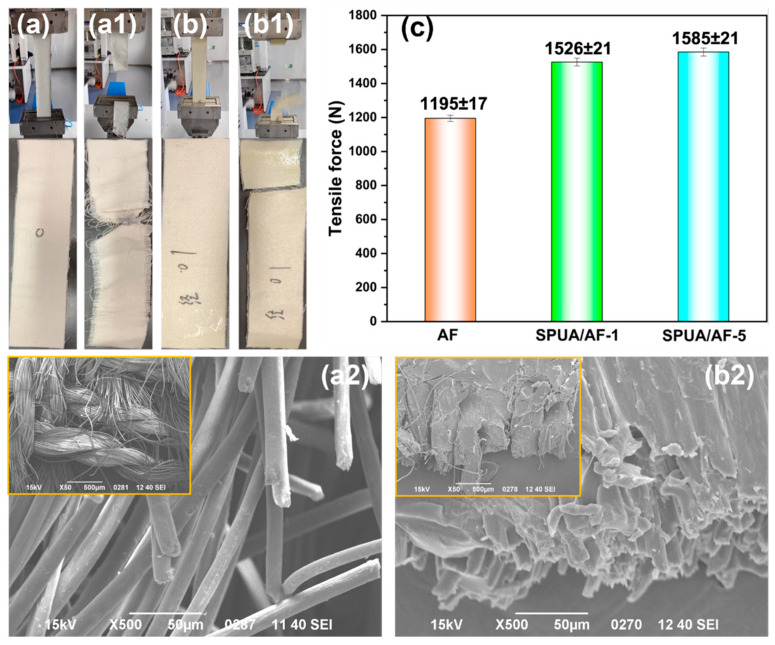
Digital photos of composite fabric before and after tensile tests: AF (**a**,**a1**), PUA/AF-5 (**b**,**b1**); The tensile force of the composite fabric samples (**c**). SEM images of AF (**a2**) and PUA/AF-5 (**b2**) after tensile tests.

The tensile force of the composite fabrics, as presented in Figure 5, clearly demonstrates the reinforcing effect of the PUA coating. The tensile force increased significantly from 1195 ± 17 N for pure AF to 1526 ± 21 N for PUA/AF-1, and further to 1585 ± 21 N for PUA/AF-5.

Visual observations during tensile testing (Figure 5(a1,b1)) revealed noticeably reduced fiber slippage and more cohesive deformation behavior in the PUA-coated fabrics, indicating enhanced interfacial adhesion and more efficient load distribution imparted by the polyurea matrix. Furthermore, SEM images of the fracture surfaces (Figure 5(a2)) show that pure AF exhibits a disordered and loosely packed fracture morphology. In contrast, PUA/AF-5 (Figure 5(b2)) displays a relatively smooth and compact fracture surface, indicative of effective stress transfer between fibers and the polyurea coating, ultimately resulting in enhanced tensile force.

**Figure 6:**
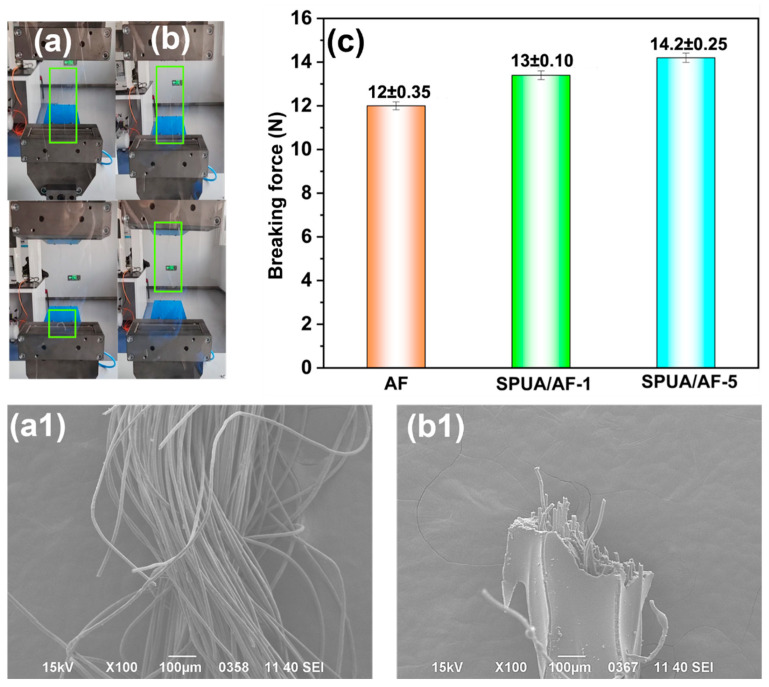
Digital photos of single yarn before and after tensile tests: AF (**a**), PUA/AF-5 (**b**); breaking force of the samples (**c**); and SEM images of AF (**a1**) and PUA/AF-5 (**b1**) after single yarn tensile tests.

Single-yarn tensile tests further confirmed the reinforcing effect of the PUA coating at the yarn scale. Digital photographs of individual yarns before and after tensile testing for both AF and PUA/AF-5 are shown in Figure 6a,b, respectively, with corresponding breaking force data summarized in Figure 6c. The tensile force of single yarns increased from 12 ± 0.35 N (AF) to 13.4 ± 0.1 N (PUA/AF-1), and further to 14.2 ± 0.25 N (PUA/AF-5).

SEM micrographs (Figure 6(a1,b1)) revealed that PUA-coated yarns exhibited more uniform fracture surfaces and stronger inter-fiber bonding, indicative of enhanced load-bearing capability and improved energy dissipation during tensile deformation. Moreover, the presence of the PUA coating significantly reduced yarn splitting and facilitated more uniform fiber elongation, further validating the positive role of polyurea in improving mechanical performance at the single yarn level.

### 3.4. Puncture Resistance and Scratch Repair Property

**Figure 7:**
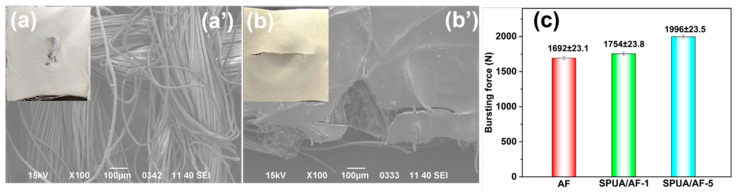
Digital photos and the corresponding SEM images of composite fabric after bursting force test: AF (**a**,**a’**), PUA/AF-5 (**b**,**b’**); bursting force of the composite fabric samples (**c**).

Bursting force tests (Figure 7) revealed significant enhancements in the mechanical robustness of the composite fabrics under concentrated load conditions. The pure AF fabric exhibited a bursting force of 1692 ± 23.1 N, while PUA-coated variants displayed increased forces of 1754 ± 23.8 N (PUA/AF-1) and 1996 ± 23.5 N (PUA/AF-5). SEM analysis of the fracture zones showed smoother fracture edges and evidence of substantial energy dissipation due to fiber–polyurea interactions, confirming the role of the polyurea coating in improving toughness and energy absorption. The observed improvement in bursting resistance is attributed to an initial viscoelastic deformation of the PUA layer, followed by a cooperative rupture process involving both the fiber network and the polymer matrix. This coordinated response effectively dissipates applied energy, illustrating the synergistic effect between the fibers and polyurea in resisting mechanical failure.

**Figure 8:**
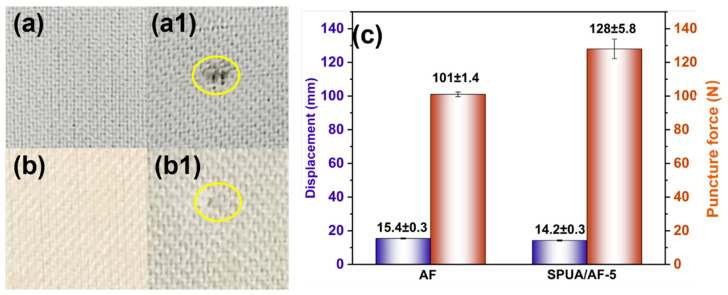
Digital photos of composite fabric before puncture: AF (**a**), PUA/AF-5 (**b**). Digital images of composite fabrics after puncture: AF (**a1**), PUA/AF-5 (**b1**). The displacement and static puncture force of the AF and PUA/AF-5 (**c**).

As shown in Figure 8, static puncture tests further confirmed the reinforcing capability of PUA coatings. Digital photographs (Figure 8(a1,b1)) highlight reduced fiber slippage and rupture, resulting from the deformation capacity of the polyurea layer, which contributed to more effective energy dissipation and improved puncture resistance. Notably, PUA/AF-5 exhibited a significantly smaller puncture hole and a higher puncture force of 128 ± 5.8 N, compared to 101 ± 1.4 N for the pure AF fabric. This enhanced puncture resistance is primarily attributed to enhanced interfacial adhesion and synchronized deformation between the fibers and the polymer coating. Additionally, puncture displacement data showed that the PUA coatings effectively limited penetration depth at the peak load, indicating improved stiffness and structural integrity of the composite under puncture stress.

**Figure 9:**
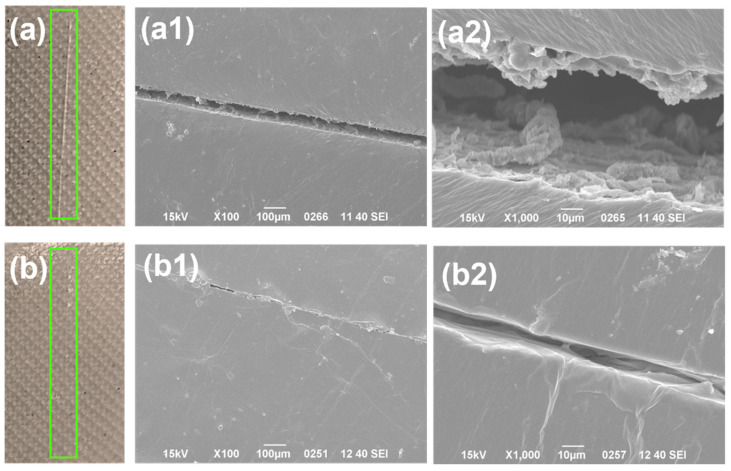
Morphological comparison of PUA/AF-5 fabric scratches before (**a**,**a1**,**a2**) and after (**b**,**b1**,**b2**) self-repair at 80 degrees for 30 min.

The scratch self-healing capability of PUA/AF-5 was evaluated through both macroscopic observation and microscopic analysis. Following thermal treatment at 80 °C for 30 min, visible surface scratches significantly diminished, as shown in Figure 9a,b, indicating effective macroscopic self-repair. SEM images further confirmed the material’s self-healing performance at the microstructural level, revealing that post-treatment scratches became substantially narrowed and partially reclosed. The self-healing mechanism is primarily attributed to the intrinsic dynamic hydrogen bonding within the polyurea matrix [25,42]. Upon thermal activation, enhanced polymer chain mobility facilitates re-entanglement and interfacial reformation across the damaged regions. This thermally induced molecular rearrangement not only repairs the polyurea network but also strengthens fiber–matrix interactions at the scratch sites. These results highlight the composite fabric’s promising self-healing functionality, which significantly improves its durability and reusability in applications requiring both puncture resistance and damage recovery.

## 4. Conclusions

In this study, the PUA/AF composite fabrics were developed, which exhibited comprehensive improvements in thermal stability, flame retardancy, mechanical properties, puncture resistance, and self-healing capabilities. TGA analyses revealed that OP550 effectively promoted char formation of the coating, enhancing the thermal protective performance of the composite fabric. Flame-retardant evaluations, including VFT, LOI, and MCC tests, confirmed that the OP550-modified polyurea coating substantially suppressed the heat release and improved char formation, effectively preventing flame propagation and significantly increasing composite fabric safety. Mechanical characterizations demonstrated that the PUA coating notably increased tearing, tensile, single yarn tensile, and bursting forces, attributed to enhanced fiber–matrix interfacial adhesion and efficient energy dissipation mechanisms during deformation. Static puncture tests further validated the composite fabric’s enhanced resistance to localized impacts, due to improved interfacial adhesion and structural integrity provided by the polyurea coating. Notably, the composite fabric exhibited outstanding scratch self-healing performance under elevated temperatures, highlighting its promising reuse potential. These integrated enhancements—flame retardancy, mechanical reinforcement, puncture resistance, and self-healing functionality—highlight the potential of PUA/AF composites as advanced multifunctional protective textiles for demanding applications, particularly where safety, durability, and material recoverability are critical.

## Figures and Tables

**Table 1:** Formulations of the composite fabric samples.

Sample	Component A		Component B				Exolit OP550 Loadings in Coatings (wt%)
	IPDI (mol)	Exolit OP550 (mol)	D2000-MIBK (mol)	D400-MIBK (mol)	E100 (mol)	E300 (mol)	
AF	0	0	0	0	0	0	0
PUA/AF-1	0.54	0	0.09	0.36	0.03	0.03	0
PUA/AF-2	0.83	0.28	0.09	0.36	0.03	0.03	21.9
PUA/AF-3	0.87	0.32	0.09	0.36	0.03	0.03	24.1
PUA/AF-4	0.91	0.36	0.09	0.36	0.03	0.03	25.9
PUA/AF-5	0.95	0.40	0.09	0.36	0.03	0.03	27.6

**Table 2:** The TGA, DTG results, and OP550 content of composite fabric samples in a N_2_ atmosphere.

Sample	T_5%_ (°C)	T_max_ (°C)	Char Residue at 800 °C (wt%)	Exolit OP550 Loadings in Coatings (wt%)
AF	387.3	461.1	51.2	0
PUA/AF-1	271.3	324.4	22.9	0
PUA/AF-2	238.1	351.1	33.4	21.9
PUA/AF-3	233.9	340.2	31.6	24.1
PUA/AF-4	227.2	340.9	33.1	25.9
PUA/AF-5	218.9	345.8	32.9	27.6

**Table 3:** LOI and VFT results of the PUA/AF composite fabrics.

Sample	LOI (%)	After-Flame Time (s)	After-Glow Time (s)	Char Length (cm) ^a^
AF	31.4	0	0	0.5
PUA/AF-1	20.9	16 ± 3	0	-
PUA/AF-2	26.5	15 ± 3	0	-
PUA/AF-3	26.9	15 ± 2	0	-
PUA/AF-4	27.4	14 ± 3	0	-
PUA/AF-5	27.8	0	0	3.2

^a^ The samples of PUA/AF-1 to PUA/AF-4 burnt to clamp without char length.

**Table 4:** MCC-related data for AF, PUA/AF-1, and PUA/AF-5.

Sample	PHRR (W g^−1^)	THR (kJ g^−1^)	HRC (J g^−1^ K^−1^)	Residue (wt%)
AF	41.1	6.4	45	37
PUA/AF-1	232.7	22.4	244	21
PUA/AF-5	144.3	14.4	145	39

## Data Availability

The raw data supporting the conclusions of this article will be made available by the authors on request.
